# A Review of the Role of Artificial Intelligence in Patients on Extracorporeal Membrane Oxygenation: A Scoping Review Study

**DOI:** 10.1002/hsr2.73118

**Published:** 2026-08-30

**Authors:** Zahra Asadi, Mahmoud Shiri Kahnouei, Fathiyeh Bahramnejad, Alun C. Jackson, Fatemeh Bahramnezhad

**Affiliations:** ^1^ Department of ICU Nursing, School of Nursing & Midwifery Tehran University of Medical Sciences Tehran Iran; ^2^ School of Advanced Technologies in Medicine, Medical Nanotechnology and Tissue Engineering Research Center Shahid Beheshti University of Medical Sciences Tehran Iran; ^3^ Department of Health Information Technology Sirjan School of Medical Sciences Sirjan Iran; ^4^ Centre on Behavioral Health University of Hong Kong Pokfulam China; ^5^ Department of ICU Nursing, School of Nursing & Midwifery, Nursing and Midwifery Care Research Center Tehran University of Medical Sciences Tehran Iran

**Keywords:** artificial intelligence, ECMO, extracorporeal membrane oxygenation, machine learning

## Abstract

**Background and Aims:**

ECMO is used to deliver cardiopulmonary support in extreme failure where standard therapies are unfruitful. Although ECMO is associated with high costs and significant risks, advances in technology and clinical management have improved its safety and patient outcomes. AI/ML may provide decision support through data analysis to alert clinicians to potential safety issues, detect complications, and help inform pump/ventilator settings. The primary objective of this scoping review is to examine the applications of AI and machine learning in patients receiving various ECMO modalities, specifically veno‐arterial (VA), veno‐venous (VV), and extracorporeal cardiopulmonary resuscitation (ECPR).

**Methods:**

We carried out a scoping review based on Arksey and O'Malley, encompassing 20 inaugural AI/ML ECMO studies (no review/theoretical/non‐English). The databases were searched till December 2025. Expressed data covered study characteristics, AI methods, inputs, objectives, performance, and limitations. Algorithm applications were categorized based on patient characteristics and clinical use (selection, timing, monitoring, weaning, and readmission). The findings were expert‐validated to identify trends and gaps.

**Results:**

Across studies (*n* ≈85,509 ECMO patients; mean per study ≈4275), AI applications were identified across selection, timing, monitoring, weaning, and readmission. Models (Random Forest, XG Boost, Light GBM, deep nets) Reported AUROCs ranged from approximately 0.70 to 1.00 for ECMO need prediction, neurological outcome, mortality, and bleeding. Imaging data were underutilized in most studies; most studies were retrospective in design. While predictive performance has been reported, direct hard outcome improvement wasn't consistently shown; AI primarily functioned as decision support.

**Conclusion:**

AI/ML are promising for predicting ECMO needs and outcomes and may support clinical decision‐making, but retrospective data and limited prospective validation temper conclusions. Prospective and real‑world validation studies are required to demonstrate clinical outcome benefits and support the integration of AI into ECMO practice and guidelines.

## 1 Introduction

Extracorporeal membrane oxygenation (ECMO) is a rescue therapy in extreme respiratory or cardiac failure when ordinary treatment fails [1]. As artificial support for the heart–lungs, ECMO provides cardiopulmonary assistance for critically ill patients with concurrent heart and lung failure [2]. Over the past decade, the global use of ECMO has expanded significantly for patients with various underlying conditions [3] and is now recognized as one of the most complex and expensive treatments in intensive care units (ICUs) and cardiac surgery [4]. Advances in clinical care, guidelines, drug discovery, and technology have reduced ECMO‐related risks and broadened its application scope to improve patient outcomes continuously [5].

Though utilized extensively, ECMO remains resource‐intensive and characterized by high complications [6]. Survivors typically acquire acute complications, disabling sequelae, and high readmission rates [7]. Given the seriousness of ECMO patients, their associated costs, and high complication rates, accurate patient selection and outcome prediction are crucial [8, 9]. Misuse of ECMO can lead to increased resource utilization, hospital spending, and death [10, 11]. Despite numerous ECMO complication and outcome prediction models in recent years, data unavailability has restricted external validation, and their predictive capacity has been reported as moderate [12].

Artificial intelligence (AI) and machine learning (ML), being advanced substitutes for traditional statistical methods, hold the potential to process complex, high‐dimensional data to find underlying patterns and interactions invisible to traditional analysis [13, 14]. The application of AI integration within ECMO technology and patient care can enhance the level of care, optimize therapy, and improve outcomes. AI algorithms can analyze data from ECMO monitors, sensors, and electronic health records and may provide early warnings for potential complications—clot formation, hemodynamic instability, or infection—and thereby support clinical decision‐making [5].

ML models can handle high‐dimensional predictors and nonlinear relationships to predict treatment and survival outcomes [11, 15, 16].

New approaches may generate more precise predictive models than traditional regression methods and may help improve blood management and critical parameters of ECMO patients [15, 16].

This scoping review includes studies involving different ECMO modalities, including veno‑arterial ECMO (VA‑ECMO), veno‑venous ECMO (VV‑ECMO), and extracorporeal cardiopulmonary resuscitation (ECPR), in order to comprehensively map current applications of artificial intelligence across the ECMO lifecycle.

## 2 Methods

The scoping review was conducted to identify, analyze, and synthesize existing studies regarding the application of artificial intelligence in critically ill patients receiving ECMO support. The review followed the guidelines provided by Arksey & O'Malley (2005) and subsequent clarifications by Levac et al. (2010).

### 2.1 Objectives and Research Questions

The primary objective of this scoping review is to examine the applications of AI and machine learning in patients receiving various ECMO modalities, specifically veno‐arterial (VA), veno‐venous (VV), and extracorporeal cardiopulmonary resuscitation (ECPR).

Questions investigated during the study were:

- At what stages of ECMO decision‐making does AI get employed?
- Which ML models (e.g., Random Forest, XG Boost, Neural Networks) have been most effective at ECMO prediction?
- What type of data (clinical, imaging, lab) has been used for training AI models, and are such data obtained retrospectively or in real‐time?
- What is the accuracy, sensitivity, and specificity of these algorithms quantitatively reported as, and are models reliable or merely supportive tools?
- Has the use of AI improved clinical outcomes such as reduced mortality, reduced ICU length of stay, or enhanced ECMO decision‐making?
- Has the integration of imaging information with clinical information maximized model performance?

### 2.2 Inclusion and Exclusion Criteria

Inclusion Criteria:

- Study Type: Original research and observational studies.
- Topic: Use of AI in predicting critically ill ECMO patient outcomes.
- Language: English‐language publications.
- Data: Quantitative or qualitative data reports from studies of AI algorithm performance.

Exclusion Criteria:

- Study Type: Theoretical articles and conference abstracts without full text, systematic reviews.
- Language: Non‐English literature.
- Data: Lack of or irrelevant data in studies.

### 2.3 Search Strategy

International databases PubMed/MEDLINE, Scopus, Web of Science, EMBASE, Cochrane Library, CINAHL, and ProQuest, and national databases Iran‐MEDEX, SID, and MAG Iran were searched exhaustively. The comprehensive search strategy, including specific syntax and Boolean operators for each database, is detailed in Supplementary File S1. The search was as of December 2025.

Boolean operators were applied to put together keywords that are also given in the appendix to make it reproducible.

Reference lists of included studies were hand‐checked, and citation tracking was also done with Google Scholar.

Authors were requested to provide full texts when not available online.

Gray literature searches were conducted through Google, checking the first 10 pages of results and further pages if needed.

### 2.4 Study Selection and Data Extraction

Titles and abstracts were independently screened by two reviewers; discrepancies were settled by discussion and consensus with a third reviewer.

Data that were extracted were:

Study characteristics: year of publication, country, design, and size.

Algorithm details: type of AI model, input features, and predictive aim.

Findings: model performance results (e.g., AUC, accuracy, sensitivity).

Limitations: algorithmic limitations and study limitations.

Minimal quality and bias assessment were carried out to ensure the reliability of findings.

### 2.5 Data Analysis and Synthesis

Data extracted from the included studies were charted and categorized according to algorithm type and patient population. In total, 20 studies were included in the review.

**Figure 1:**
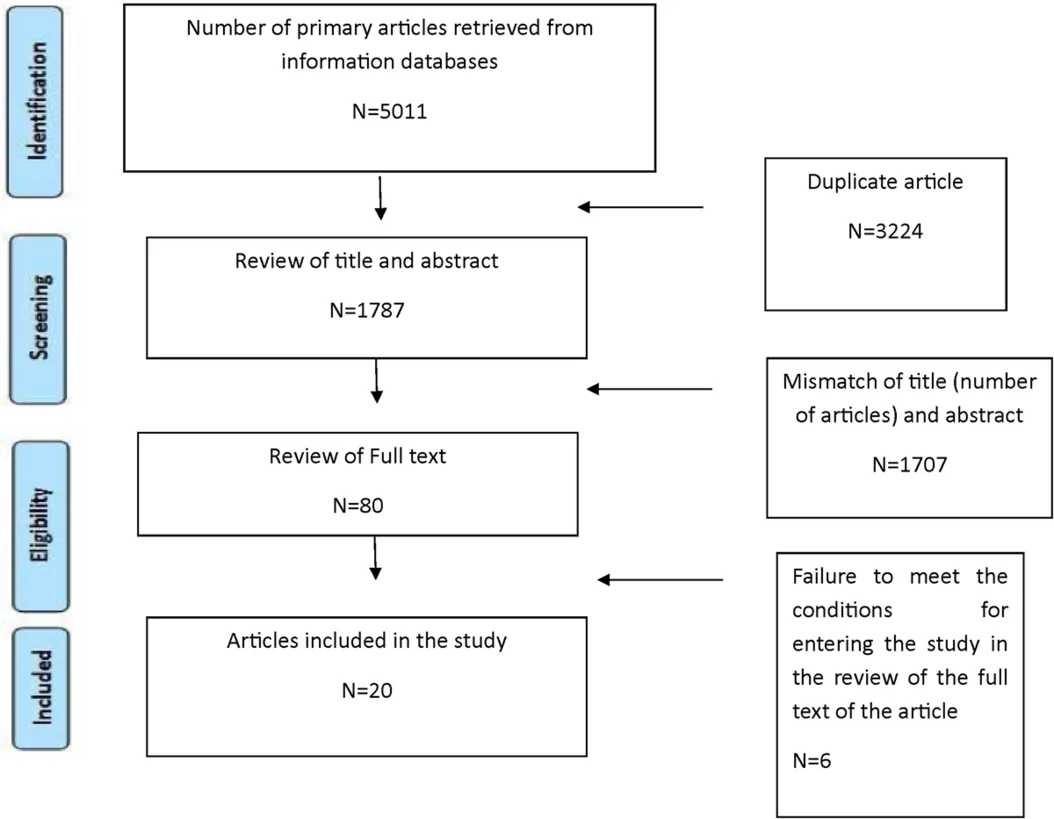
Entry of studies into the review.

This scoping review was conducted and reported in accordance with the Preferred Reporting Items for Systematic Reviews and Meta‑Analyses extension for Scoping Reviews (PRISMA‑ScR) As illustrated in Table 2, the majority of the included studies (90%) demonstrated moderate to high methodological quality, with only one study categorized as low quality due to its limited sample size. The study selection process is presented in the PRISMA flow diagram (Figure 1), and the characteristics of the included studies are summarized in Table 1.

**Table 1:** Characteristics of studies included in the scoping review.

First author	Objective	Research design	Sample size	Type of AI used	Conclusion
Louis Morisson[17]2023 *Paris, France*	to develop and validate a machine‐learning algorithm to predict the need for VA‐ECMO implantation in patients with PC‐LCOS	retrospective study	190 patients	Pediatric ECMO Quantification machine learning‐enabled AI	Ensemble machine learning algorithm helps clinicians predict risk of deterioration and need for VA‐ECMO implantation in moderate to severe PC‐LCOS patients
Andrew F. Stephens [18] 2023 *worldwide*	Using a large international patient cohort to develop and validate an artificial intelligence‐based tool to predict in‐hospital mortality from VA‐ECMO	retrospective cohort	18,167 patients	A deep neural network, ECMO Predictive Algorithm (ECMO PAL)	The developed AI‐based model had high generalizability for predicting in‐hospital mortality in patients undergoing veno‐arterial ECMO treatment.
Shuo Wang [19] 2024 *Beijing, China*	to clarify the wide mortality variance observed in refractory CS, attributing it to the condition's inherent heterogeneity	a single‐center, observational study	210 patients	Consensus k‐means algorithm analysis	This machine learning was used to investigate the heterogeneity among CS patients treated using VA‐ECMO and analyzed their clinical, biological, and inflammatory profiles to classify them into unique sub phenotypes. These classifications correlate with specific clinical characteristics and mortality rates, underscoring the importance of early identification.
Adeel Abbasi [20] 2020 *IRAN*	Accurately predict bleeding and thrombosis risks and identify new factors that are not clinically predicted or identified by traditional biostatistical methods using machine learning	cohort	44 consecutive patients	compared chi‐square to five supervised classifcation and regression machine learning models	Machine learning was used to predict ECMO complications, and the decision tree model predicted bleeding with promising accuracy, despite the small sample size.
Shuai Wang [12] 2025 *Hangzhou, China*	to develop an economical, rapid, high‐precision, and highly generalizable model for predicting the risk of death within 28 days after VA‐ECMO weaning based on easily accessible patient characteristics	A multicenter, retrospective cohort study	225 patients	This study developed and evaluated 10 classical and advanced ML models to predict the mortality risk of VAECMO patients,	It has the potential to significantly enhance clinical decision‐making, helping doctors better assess patient prognosis, optimize treatment plans, and improve survival rates.
Haeun Lee [21] 2023 *South Korea*	to develop ML‐based models for 90‐day and in‐hospital mortality prediction in patients treated with VV‐ECMO	retrospective observational cohort study	368 patients with acute respiratory failure undergoing VV‐ECMO	Six supervised ML models based on regression, tree ensembles, gradient boosting and neural networks were trained using 10‐fold cross‐validation to predict 90‐day mortality in patients who underwent VV‐ECMO	The ML prediction model for 90‐day mortality could accurately identify VV‐ECMO candidates with a low probability of success and could provide valuable prognostic information
Ruben Crespo‐Diaz [22] 2024 Minnesota	to create a selection model using machine learning (ML) tools for refractory cardiac arrest patients undergoing ECPR	Retrospective cohort study	376 patients		This model allows customization of patient selection to fit varied acceptable risk profiles depending on the available resources of any specific ECPR program
Tae Wan Kim [23] 2023 *Seoul, South Korea*	To utilize ML methodologies to identify critical factors that can influence neurological prognosis following ECPR	a retrospective, single‐center, observational study	330 patients	Using Shapley's Additive explanations, critical variables were first identified, which were then incorporated into machine learning analyses. Eight machine learning algorithms were trained and tested on a binary classification task involving neurological outcomes of survivors after ECPR.	machine learning methods showcased outstanding predictive accuracy for poor neurological outcomes in patients who underwent ECPR
Albert Leng [24] 2024 *Baltimore, Maryland, United States.*	to use machine learning (ML) algorithms trained on rich granular flowsheet data from electronic medical records (EMR) to identify “modifiable” risk factors associated with adverse neurological outcomes in VV‐ECMO.	retrospective review	99 patients	f 4 ML algorithms in predicting neurological outcomes: Random Forest, CatBoost, LightGBM and XGBoost	developed the framework for the first prognostic model for predicting neurological outcomes after VV‐ECMO cannulation.
Tadashi Kamio[25]2025 *Japan*	to develop and validate an ML algorithm to predict bleeding complications	retrospective cohort study	357 patients	The LightGBM and Random Forest models	This tool can support early identification of high‐risk patients and improve overall transfusion management
Joshua Fuller [26] 2024 *Columbia*	to assist clinicians with the decision to decannulate a patient from ECMO	retrospective chart review	118 patients	Continuous Evaluation of VV‐ECMO Outcomes (CEVVO)	CEVVO exhibited improved performance on synthetic data with inherent temporal dependencies (*p* < 0.001) compared with logistic regression and a dense neural network.
Andrew Kalra [27] 2023 *Baltimore, Maryland, United States.*	to use ML to predict ABI^1^ and to identify associated risk factors using the largest ELSO^2^ database of adult VV‐ECMO patients.	retrospective study	37,473 patients	Random Forest, CatBoost, LightGBM, and XGBoost	Performance was sub‐optimal due to the low reported prevalence of ABI with lack of standardization of neuromonitoring/imaging protocols and data granularity in the ELSO Registry.
kepeng liu [28] 2023 *Data were collected from the BioStudies SITE*	To explore whether machine learning based on 5 algorithms could be used to predict severe hemolysis in critically ill patients undergoing adjuvant ECMO therapy.	retrospective analysis	1063 patients	XGB	According to our results, performed best overall, with an AUC > 0.8, an accuracy > 90%. Besides, the top 5 factors found to influence hemolysis by data analysis
Peta M Alexander [29] 2022 *USA*	to predict clinically significant bleeding in the ECMO‐supported population		272 patients	graph neural network	EHR^3^‐derived AI‐prediction models of bleeding in this complex patient population are at least as accurate as models with manual data and traditional statistical analysis
Julia Braun [30] 2023 *Zurich, Switzerland*	to provide an easy‐to‐use, pencil‐and‐paper algorithm using the machine learning technique called unbiased recursive partitioning, based on conditional inference trees to develop and validate a prognostic model that can predict the probability of patient survival before the initiation of ECMO therapy.	retrospective	837 patients	Conditional inference trees	In approximately 64% to 68% of cases, the prediction based on pre‐ECMO variables matched the observed outcome,
Jeffrey Balian [31] 2024 *USA*	directing efforts for quality improvement, prior work has sought to delineate clinical factors associated with nonelective rehospitalization following ECMO	Retrospective cohort study	22,947 patients	XGBoost	In the present work, ML models exhibited superior accuracy in evaluating nonelective readmission, compared with LR.
Benjamin L. Shou [32] 2025 *Stanford, California*	to incorporating rich electronic medical record (EMR) data to predict neurological outcomes after venoarterial extracorporeal membrane oxygenation (VA‐ECMO).	retrospective review	194 patients	electronic medical record (EMR), XGBoost	An interpretable machine learning model from EMR‐extracted data was able to predict neurological outcomes for patients undergoing VA‐ECMO with excellent accuracy.
K P Kresoja [33] 2024 *Germany*	To identify the impact of VA‐ECMO therapy on outcomes by leveraging the potential of machine learning.	randomized controlled trial	209 patients	XGBoost	the influence of VA‐ECMO therapy on 30‐day mortality, even when investigated with complex machine learning models, is negligibly low
Hsu, Jung‐Chi [34] 2025 *Taiwan*	to develop and validate an ML‐based mortality prediction for cardiac support ECMO patients, delving deeper into the multifaceted dynamics influencing ECMO patient outcomes.	derivative cohort	1748 eligible patients	Machine learning with the random survival forest (RSF) model	machine learning can help identify high‐risk populations for tailored management
Brian Ayers [35] (2020)	To develop a machine learning algorithm to augment clinical decision making related to VA‐ECMO.	retrospectively	282	Deep Neural Networks	the final model is showing an overall accuracy of 82% and an area under the curve (AUC) of 0.92 in the experimental group, meaning that the model's power in distinguishing between living and deceased patients is very high, and is a strong indication of its reliability.

1 Acute brain injury.

2 Extracorporeal Life Support Organization.

3 Electronic health record.

Descriptive analyses were performed, including frequency distributions of key variables and study characteristics.

To assess the methodological quality and risk of bias of the included observational studies, the Newcastle‐Ottawa Scale (NOS) was employed. This tool evaluates studies across three domains: Selection (maximum four stars), Comparability (maximum two stars), and Outcome (maximum three stars), yielding a total score ranging from 0 to 9 stars. Two reviewers independently appraised each study using the NOS checklist. Based on the aggregate scores, the quality of studies was categorized as high (8–9 stars), moderate (5–7 stars), or low (0–4 stars). Any disagreements between the reviewers were resolved through discussion and consensus with a third senior reviewer. The detailed quality assessment results for each included study are presented in Supplementary File S2.

### 2.6 Stakeholder Consultation

Two experts in AI and critical care medicine were consulted.

Their feedback was incorporated into data interpretation and analysis to ensure agreement with research and clinical requirements.

### 2.7 Ethical Consideration

This article is a scoping review that synthesizes data from previously published studies available in the public domain. No new data were collected from human participants or animals for the purposes of this review. Therefore, formal approval from an Institutional Review Board or Ethics Committee was not required. The authors confirm that the review was conducted in accordance with recognized ethical standards in research and scientific reporting, including honest and transparent presentation of the evidence, appropriate citation of all sources, and avoidance of plagiarism.

## 3 Results

In total, 20 studies were analyzed with sample sizes ranging from 44 to 37,473 patients, encompassing a total of 85,509 ECMO patients. As illustrated in Supplementary File S2, the majority of the included studies (90%) demonstrated moderate to high methodological quality, with only one study categorized as low quality due to its limited sample size.

**Figure 2:**
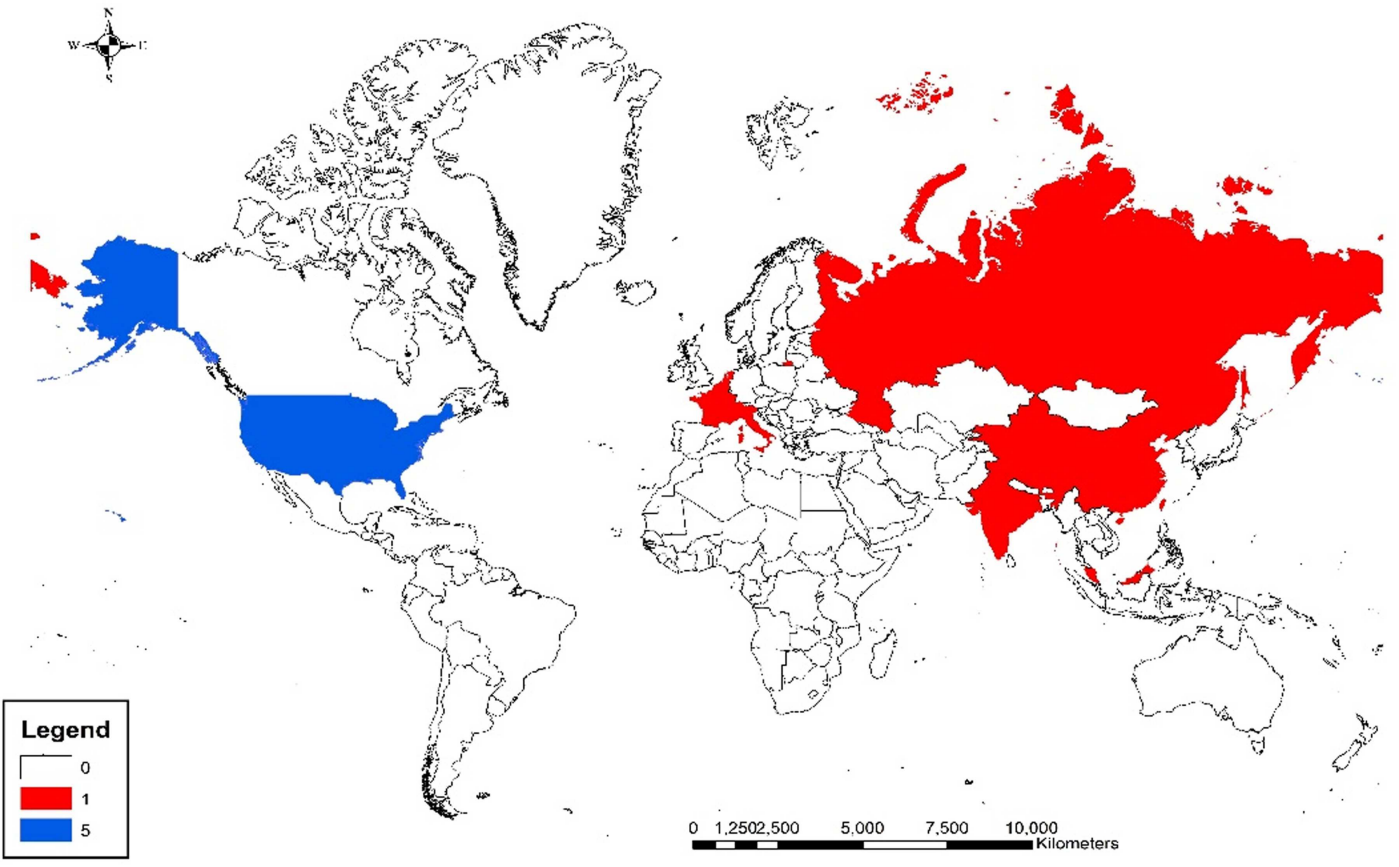
Geographical distribution of countries participating in the study.

“Sample sizes averaged 4275 but showed high heterogeneity between single‐center and database‐driven studies, impacting model generalizability. Notably, over 50% of the research originated from the United States [2]. Included studies spanned various modalities, including VA‐ECMO, VV‐ECMO, and ECPR, alongside mixed registry‐based datasets. Geographically, while research is a globally expanding field, hotspots are primarily concentrated in the United States and China (Figure 2). Key findings addressing our research questions are summarized below:

### 3.1 At What Stage of ECMO Decision‐Making Is AI Being Used?

AI is utilized throughout the ECMO continuum—from necessity prediction and initiation timing to real‐time monitoring, complication forecasting, and weaning. Current applications are categorized into three primary domains:

A. **Pre‐ECMO Initiation (Evaluation and Selection)**
  In this phase, AI optimizes candidate selection and timing through:
  A.1 Necessity and Mortality Prediction: Algorithms (e.g., Conditional Inference Trees) estimate pre‐intervention mortality to identify patients most likely to benefit from therapy [17, 36, 37].
  A.2 Treatment Response and Timing: Models predict clinical responses to guide optimal initiation, especially in critically ill cohorts [18].
  A.3 Risk Assessment without Early Intervention: AI forecasts risks like mortality or neurological injury in the absence of early ECMO, supporting proactive decision‐making [19].
B. **During ECMO therapy (Monitoring, outcome prediction, and complication management)**
  Most reviewed studies focus on this phase, utilizing models like Random Survival Forests (RSF), Decision Trees, and Neural Networks for:
  B.1 Mortality Risk: Predicting in‐hospital mortality by integrating clinical and laboratory data [11, 19, 20, 21, 22, 23].
  B.2 Acute Brain Injury (ABI): Forecasting ABI risks to guide neurological management [24].
  B.3 Hemorrhagic/Hemolytic Complications: Analyzing coagulation and blood profiles to personalize anticoagulation and prevent hemorrhagic shock [23, 25, 26, 27].
  B.4 Neurological Outcomes: Predicting outcomes specifically in ECPR cohorts to inform decisions regarding care continuation or withdrawal [17, 28, 29].
C. **Weaning and Post‐Discharge Follow‐up**
  AI optimizes recovery transitions through:
  C.1 Weaning Optimization: Analyzing physiologic trajectories to supplement clinical judgment in determining the ideal weaning window [30].
  C.2 Readmission Risk: Predicting 90‐day post‐discharge readmission to enhance long‐term care planning [31].

AI and Machine Learning (ML) provide essential decision support across the ECMO continuum—from candidate selection to post‐discharge care. By enhancing monitoring, complication management, and resource optimization, these tools demonstrate broad potential to improve survival and clinical precision in advanced critical care.

### 3.2 Which Machine Learning Models (e.g., Random Forest, XGBoost, Neural Networks) Have Been Most Effective at Predicting the Need for ECMO?

Several studies identified XG Boost as the most powerful boosting model in external and internal validations.

For instance, XG Boost yielded ROC AUC = 0.854 (external) and 0.857 (internal) in a study, demonstrating high discriminative performance for VA‑ECMO requirement prediction [19]. In another study comparing seven algorithms (Decision Tree, Logistic Regression, Random Forest, Ada Boost, SVM, and DNN), the deep neural network (DNN) performed best in in‐hospital mortality prediction [20].

For identifying cardiogenic shock patients on ECMO, k‐means consensus clustering in combination with a deep super learner (DSL) that ensembles Decision Tree, Random Forest, XG Boost, and Neural Network modules showed optimal performance [18]. In predicting bleeding and thrombosis, models such as Random Forest, Decision Tree, k‐NN, and Logistic Regression were compared on the basis of recursive feature elimination and chi‐square testing. Results indicated higher accuracy for the prediction of bleeding than thrombosis, with anticoagulation variables as significant predictors [25].

Another study identified Random Forest as the best model to predict 28‐day mortality after ECMO, with AUROC = 1.00 and 0.97, suggesting that the model may capture complex relationships within the dataset [11].

Boosting models, XG Boost and Light GBM, also performed extremely well to predict ECMO requirement—XG Boost with AUROC = 0.82 (internal) and 0.75 (external) [37]. Random Forest accurately predicted neurological outcomes after ECPR, with AUC = 0.89 (training) and 0.80 (validation) [17].

Of 8 algorithms, Logistic Regression, Random Forest, and XG Boost were the 3 best performers, while SVM and MARS had poor performance [28].

Comparison among Random Forest, XG Boost, Light GBM, and Cat Boost found no difference among them [32].

In another comparison, Light GBM and Random Forest were the best in ECMO requirement prediction, outperforming SVC and XG Boost. The CEVVO model was superior to Neural Networks and Logistic Regression in ECMO weaning success prediction [27, 30].

XG Boost had the highest AUC ≈ 0.70 in ABI prediction, though its positive predictive value (15%) was a drawback [24].

For the prediction of severe hemolysis, XG Boost AUC = 0.913 performed better compared to other models [11].

Comparing graph neural networks (GNN) and Logistic Regression, there was no difference in performance, but both were effective in ECMO necessity prediction [33].

Conditional Inference Trees also predicted mortality and pre‐ECMO risk with high accuracy maintained, despite reductions in performance during external validation [36].

XG Boost was found to perform better compared to Logistic Regression for post‐ECMO readmission prediction [31].

In predicting neurological outcome, XG Boost with EMR features and Shapley explanations achieved AUROC = 0.90 [29].

Another study for 30‐day mortality reported AUC = 1.00 (training) and 0.80 (validation) [34]. The Random Survival Forest (RSF) model achieved AUC = 0.953 in predicting ECMO mortality, outperforming traditional scoring systems such as SOFA and APACHE II [21]. Finally, Deep Neural Networks performed with 82% accuracy and AUC = 0.92 in predicting survival, which was significantly better than the SAVE score (AUC = 0.65) [22].

### 3.3 What Type of Data (Lab, Imaging, Clinical) Were Employed for Training the AI Algorithms, and Were They Prospectively or Retrospectively Recorded?

The reviewed AI models primarily utilized retrospective clinical and laboratory data, including physiological parameters (e.g., arterial lactate), risk scores (e.g., Euro SCORE II), and occasionally time‐series data. While most studies relied on retrospective cohorts, prospective real‐time data integration remains limited [19]. Patient data in the Extracorporeal Life Support Organization (ELSO) international registry were used retrospectively for model training in another study [20].

Comprehensive datasets were retrospectively compiled to train machine learning models, encompassing longitudinal biochemical profiles (CBC, organ function, inflammatory markers, and ABGs), hemodynamic parameters (MAP, LVEF, and ECMO flow), demographic data, and pharmacological interventions [11].

Clinical data—patient demographics, symptoms, ECMO indications, and related complications like bleeding and thrombosis—were drawn from extant medical records and retrospectively utilized to train the AI models [25].

Other studies utilized 25 clinically accessible variables regarding patient examination (age, body mass index, respiratory status, ECMO indications) and laboratory data (anti‐factor Xa and thromboelastographic), collected and analyzed retrospectively [11].

Training datasets were categorized into two main groups: structured Electronic Health Record (EHR) data (comprising ~40 variables such as vitals and disease severity) and manually recorded clinical features (~51 variables including granular bedside monitoring and laboratory data), both primarily captured around ECMO initiation [37].

Models were retrospectively trained using per‐arrest parameters, including initial rhythm, iROSC, airway management duration, and metabolic profiles (pH, ABGs, and lactate levels) [17]. Baseline clinical data (comorbidities, behavioral risk factors, ICU management parameters, mean arterial pressure in the first 24 h) and laboratory parameters documented between 6 h before ECMO initiation were retrospectively incorporated in some studies [28].

Retrospective data encompassed demographics, clinical outcomes, and longitudinal trajectories (slopes and variability) of vitals, lab results, and ECMO/ventilator settings captured pre‐ and post‐cannulation [32].

Clinical information—vital signs, labs, primary and discharge diagnoses, and comorbidities—and hospital procedural information (e.g., acute dialysis treatment, ECMO treatments, medications) were obtained retrospectively [27]. AI models integrated discrete retrospective clinical data with continuous real‐time telemetry from ECMO devices, encompassing hemodynamics (e.g., BP, SpO2) and technical pump parameters [30]. A second investigation included 65 clinical parameters assessed before and during ECMO support, including age, sex, risk factors, and indication for ECMO, all of which were retrospectively extracted from the ELSO registry [24]. To predict severe ECMO‐related hemolysis, models retrospectively utilized biomarkers captured near treatment initiation, including inflammatory (CRP, cytokines), coagulation (aPTT, platelets), and hemolytic (LDH, FHB) markers [23]. EHR records of data on ECMO‐related clinical events, therapeutic interventions, bleeding complications, and other treatment‐related events were retrospectively used for AI model development, too [33]. Models were retrospectively trained on clinical demographics, indications (e.g., postcardiotomy, cardiogenic shock), and biochemical markers (lactate, e‐GFR, albumin), notably lacking integration of imaging modalities such as CT, MRI, or ultrasound [36].

Retrospective data for adult ECMO patients (≥ 18 years) were extracted from healthcare databases, encompassing ICD‐10 diagnoses, Elixhauser comorbidity indices, and multi‐systemic intra‐ and postoperative complications [31].

Electronic medical record data (vital signs, labs, and ECMO and ventilator settings) were collected 24 h before cannulation through the completion of the ECMO course retrospectively. No imaging data were used within this study [29]. In a different study, the emphasis was on clinical features of ECMO patients, i.e., age (mean 63 years), male percentage (81%), and ST‐elevation myocardial infarction (STEMI; 67%), which were applied to train an XGBoost model [34]. Clinical data—such as age, physiological status, lab values, complications, and disease severity scores such as SOFA and APACHE II scores—were mostly recorded at admission or during ECMO therapy and used for constructing machine learning models [21].

Models utilized ~270 EMR‐derived variables (including lactate, bilirubin, and creatinine) captured within the first 48 h of VA‐ECMO; however, no imaging data (e.g., echocardiography) were integrated [22].

### 3.4 What Was the Accuracy, Sensitivity, and Specificity of the Algorithms Reported? Are the Models Reliable or Largely Supportive?

Using a probability cut point of 0.168, the Deep Super Learner (DSL) algorithm had a sensitivity of 89.5% (range: 73.7%–100%), specificity of 63.7% (56.4%–70.8%), precision of 21.5% (12.8%–31.3%), and negative predictive value (NPV) of 98.2% (95.6%–100%) in the external validation data set [19].

Models were trained on ~270 EMR variables from the first 48 h of VA‐ECMO, excluding imaging data. Internal validation yielded 75.5% accuracy (Sensitivity: 82.1%; Specificity: 77.6%). In external validation (*n* = 5015), performance remained robust with 72.7% accuracy, showing minimal degradation (< 3%). Performance was consistent across indications, peaking in toxin‐associated cases (85.7% sensitivity) and lowest in myocarditis [20].

Light GBM and Random Forest models demonstrated moderate discriminative performance (Accuracy: ~70.5%; AUC > 0.70). However, the absence of reported sensitivity/specificity and the need for prospective real‐world validation position these models currently as only supplementary clinical tools [18].

Models showed moderate accuracy for bleeding (58%–80%) but poor performance for thrombosis (40%–64%). The omission of sensitivity and specificity data precludes a definitive assessment of their clinical reliability [25].

Random Forest (RF) exhibited near‐perfect internal performance (AUROC: 0.93–1.00), with 100% sensitivity and specificity in initial validation sets—metrics strongly suggestive of overfitting. While RF outperformed the weaker KNN model, these “flawless” results require cautious interpretation and external validation before clinical adoption [11].

Random Forest (RF) achieved near‐perfect metrics (AUROC: 0.93–1.00), though such flawless performance in small cohorts strongly suggests overfitting. In larger comparisons, Light GBM and XG Boost demonstrated more realistic discriminative power (AUROC: 0.79–0.83), significantly outperforming traditional PRESERVE and RESP scores. Despite lacking reported sensitivity/specificity in some models, these algorithms—particularly XG Boost—show promise as clinical decision‐support tools rather than replacements for expert judgment [37].

Another model demonstrated adequate discrimination (AUC: 0.89 training vs. 0.80 validation), with misclassification analysis supporting reproducible individual‐level performance. Given their moderate reliability, these models are currently best utilized as clinical adjuncts rather than standalone decision‐making tools [17].

For neurological outcome prediction, XG Boost emerged as the leading model (Accuracy: ~73.9%; AUC: 0.837; Sensitivity: ~70%; Specificity: ~74%). Despite this moderate discriminative power, these metrics necessitate interpreting AI outputs as clinical adjuncts rather than replacements for expert judgment [28].

XG Boost achieved the highest performance (AUC: 0.87; Accuracy: 0.80; Sensitivity: 0.81; NPV: 0.86), demonstrating promising predictive ability. Further real‐world validation is warranted before adoption as a clinical decision‐support tool [32].

Accuracy of approximately 70.5%, sensitivity ≈78.4%, and AUROC > 0.7 were found for Light GBM and Random Forest models in different studies.

These parameters demonstrate moderate‐to‐good performance and may represent potentially useful adjunctive tools in clinical practice [27].

CEVVO models demonstrated acceptable but moderate performance (mean AUROC: 0.69; mean AP: 0.86), with improved predictive power over logistic regression, decision trees, and DNNs in specific time windows [30]. Models exhibited poor predictive performance for VV‐ECMO‐related acute brain injury (ABI) (AUC: 0.67; Accuracy: 79%; Sensitivity: 39%), suggesting a need for data enrichment and improved protocols to enhance reliability [24]. XG Boost achieved the best performance (Accuracy > 90%, AUC > 0.8), with top validation AUCs of 0.806, 0.781, and 0.759. However, the lack of sensitivity and specificity reporting hinders full evaluation, despite potential efficacy in identifying high‐risk patients [23].

Previous studies reported cross‐validation error rates of 35%–36% and internal AUCs of 0.70–0.71, which dropped to 0.60–0.63 upon external validation. A Brier score of 0.21 suggests moderate accuracy, indicating these models are best used as ancillary tools, not standalone decision‐making systems [36].

The XG Boost model showed better discrimination than logistic regression (LR), AUROC = 0.64 versus 0.49 (*p* < 0.001), though overall moderate performance was observed.

Mean average precision (m‐AP) and Brier score were reported, but not quantitatively detailed [31].

AUC ranged in a series of models from 0.72 to 0.90, showing favorable performance in predicting neurological outcome [29].

In a different study, the XG Boost model sensitivity = 95% (88%–100%), specificity = 55% (40%–69%), PPV = 65%, NPV = 92%, and AUC = 0.80. This shows good ability in the detection of dead patients, but poor accuracy in the detection of survivors [22].

### 3.5 Has There Been an Improvement in Clinical Outcomes With AI Use? for Example, Reduced Mortality, ICU Length of Stay, or Reduced ECMO Decision Time?

AI and machine learning are increasingly used in critical care for ECMO therapy, with numerous studies evaluating models for VA‐ECMO need prediction and outcomes. While models like Deep Super Learner show promise in risk prediction and feature identification, their direct impact on clinical outcomes (e.g., mortality, ICU stay, decision time) remains unreported [19].

The ECMO PAL model was also better at predicting VA‐ECMO mortality, with favorable accuracy and sensitivity, compared to traditional scoring systems. It has the potential to support risk stratification and therapeutic decision‐making [11, 20].

Besides, models have been built to predict 90‐day mortality in VV‐ECMO patients and identify candidates with poor success prospects and maximize ECMO resource utilization, although no direct evidence of clinical outcome improvement thereof has been reported [37].

Although recent research has not established the direct clinical benefits of AI, the development of the models can lead to faster and more accurate assessments in patient evaluation, may assist clinicians in decision‐making; however, evidence that they decrease mortality or improve neurological outcomes remains limited [17].

Machine learning identified serum lactate and ECMO pump‐off time as key predictors of poor neurological outcomes, demonstrating the accuracy and clinical utility of these tools for neurological outcome prediction [28]. Machine learning studies (e.g., Light GBM, Random Forest) predicting ECMO bleeding complications have focused on model development and testing, neglecting direct clinical outcome enhancement [27, 32].

Other algorithms, such as CEVVO, have also been part of patient risk stratification and prediction of successful weaning from ECMO, which may potentially enable more timely decision‐making and indirectly enhance clinical outcomes [30]. On the other hand, electronic medical record (EMR) data machine learning algorithms have been reported to be very accurate for predicting neurological outcomes of VA‐ECMO patients, although no direct effect on mortality reduction or length of stay in the ICU has been reported [29].

While machine learning algorithms show impressive prediction accuracy for VA‐ECMO, their therapeutic effect on 30‐day mortality is minimal, with clinical variables playing the most significant role in final outcomes [34]. Further, models such as XG Boost have been found to outperform traditional models for the identification of high‐risk readmission of patients to the hospital [31].

Overall, studies utilizing AI in ECMO contexts show no strong evidence of improving immediate clinical outcomes (e.g., mortality, ICU stay, decision timing). Most research focuses on model validation and prediction, rather than outcome enhancement [21, 22, 36].

While studies report favorable predictive performance (AUROC 0.70–1.00), most models use retrospective data and observational settings, limiting current evidence to predictive capability rather than proven improvements in patient‐centered outcomes.

### 3.6 Has Model Performance Improved With Integration of Imaging Data (e.g., Echocardiography) With Clinical Data?

Our study focused on developing machine learning algorithms to predict VA‐ECMO need in PC‐LCOS patients, primarily using clinical and laboratory variables (e.g., lactate, VIS, Euro SCORE), without incorporating imaging data like echocardiography [19]. Echocardiography and imaging information were not included in the data sets used; therefore, the effect of combining imaging and clinical information on model performance was not assessed [20].

Most of the data employed were clinical, laboratory, and biochemical information obtained from medical records and blood samples [18].

Similar studies have also used mostly clinically recorded data, and imaging data, such as echocardiography, have not been reported to be incorporated in model development or testing [17, 21, 31, 34, 37].

These studies were primarily centered on serum markers such as lactate level, blood pressure, and ECMO phase‐specific variables, but did not include imaging data in clinical datasets [28, 30].

In other research, clinical, laboratory, and vital sign data as time‐series inputs before and after cannulation were used, while imaging data, particularly echocardiography, were not directly employed in the models [24, 32]. For the forecasting of ECMO‐associated complications, such as bleeding, imaging data were not considered or included [32, 33].

Generally, integration of imaging and clinical data was not considered in the studies compared; therefore, a final decision could not be drawn regarding how integration of the two types of data may influence predictive model performance [22, 23, 36] (Table 2).

**Table 2:** Summary of results on the application of artificial intelligence before, during, and after ECMO.

Stage	Application of artificial intelligence	ECMO Modality	Machine learning models/Algorithms	Data used	Accuracy, sensitivity, specificity	Clinical impact	Remarks
**Before ECMO** [17, 18, 19, 21, 22, 30]	Patient selection, prediction of ECMO requirement, decision‐making regarding initiation timing	Mostly VA‐ECMO and Mixed ECMO populations	Conditional inference trees, Random Forest, XGBoost, Deep Neural Network	Clinical, laboratory, and demographic data	AUROC: 0.82–0.857; Accuracy: 75%–85%; Sensitivity: 70%–89%	Indirect improvement in decision‐making time and risk stratification	Prediction of mortality, neurological outcomes, and identification of high‐risk patients
**During ECMO** [12, 17, 18, 20, 22, 24, 25, 27, 28, 29, 32, 33, 34, 35]	Patient monitoring, prediction of mortality, acute brain injury (ABI), bleeding, hemolysis, neurological outcomes	VA‐ECMO, VV‐ECMO, and Mixed ECMO populations	Random Survival Forest, XGBoost, LightGBM, CatBoost, Deep Neural Network	Clinical, laboratory, physiological, and time‐series data	AUROC: 0.7–0.953; Accuracy: 72%–92%; Sensitivity: 39%–95%	Support for treatment management, guidance of anticoagulant therapy, identification of neurologically high‐risk patients	Prediction of mortality, bleeding, hemolysis, ABI, and neurological outcomes
**After ECMO** [26, 31, 32]	Decision‐making on ECMO weaning timing, prediction of readmission	Mostly Mixed ECMO populations	XGBoost, CEVVO, Random Forest	Clinical, laboratory, and electronic medical record (EMR) data	AUROC: 0.64–0.87; Accuracy: 70%–80%; Sensitivity: 55%–95%	Assistance in follow‐up planning and prediction of successful ECMO weaning	Indirect improvement, better patient stratification

## 4 Discussion

This scoping review provides a comprehensive overview of the current landscape of AI applications in the management of patients undergoing ECMO. However, the overall body of evidence remains dominated by retrospective, single‐center cohort studies with heterogeneous endpoints and variable reporting standards, which collectively limit the strength and generalizability of the conclusions that can be drawn.

The predominance of retrospective designs in this field is not surprising, given the relative rarity of ECMO and the logistical challenges of conducting prospective trials in critically ill populations. This ‘indication bias's is particularly problematic for pre‐ECMO prediction tools, which aim to guide the critical decision of whether to initiate ECMO support.

The moderate to favorable predictive performance reported across the included studies suggests that AI holds promise as a decision‐support tool in ECMO management. However, several important considerations temper the optimism that might be inferred from numerical performance metrics alone [8, 9, 12, 18, 21, 26, 28, 29, 32, 33, 34, 35, 37]. First, the heterogeneity in model inputs and outcome definitions across studies complicates meaningful comparison of model performance. While some models focused on mortality prediction, others targeted specific complications such as acute kidney injury, neurological events, or successful weaning. This variability reflects the diverse clinical challenges in ECMO care but also means that ‘performance’ is not a monolithic concept—a model that excels at predicting mortality may not necessarily generalize to predicting weaning success.

The field would benefit from consensus on core outcome sets and standardized reporting guidelines for AI studies in ECMO, similar to the TRIPOD statement [38, 39, 40]. Second, the consistently high performance metrics reported in several studies, particularly those approaching near‐perfect discrimination, warrant careful scrutiny. Such values in relatively small, single‐center cohorts are highly suggestive of overfitting and optimism bias.

When a model fits the idiosyncrasies of its training data too closely, it captures noise rather than signal, leading to inflated performance estimates that are unlikely to be replicated in independent cohorts. The ‘validation gap’—the disconnect between excellent retrospective performance and unproven prospective utility—remains one of the most critical barriers to clinical translation.

### 4.1 Type of Data

Most artificial intelligence models developed for ECMO populations were trained using multimodal clinical datasets, commonly incorporating demographic characteristics, comorbidities, vital signs, and laboratory parameters. However, recent advances in deep learning for medical image analysis suggest that these barriers are surmountable, and future research should prioritize the development of multimodal models that combine imaging with clinical and laboratory data. The underutilization of high‐frequency physiological monitoring data represents another evidence gap [41, 42, 43, 44, 45, 46, 47, 48]. Echocardiography provides dynamic, real‐time information on ventricular function, valvular competence, and cannula positioning that is critical for weaning decisions and complication detection. Similarly, neuroimaging could offer invaluable insights into neurological injury, a major cause of morbidity in ECMO patients. The rarity of imaging integration likely reflects technical barriers: the lack of standardized acquisition protocols, the absence of automated feature extraction pipelines, and the computational challenges of processing unstructured video data. The transition from static ‘snapshot’ models to dynamic, continuous‐learning systems that incorporate real‐time waveform data would represent a paradigm shift, enabling not just risk stratification but truly actionable, time‐sensitive clinical decision support.

However, for these models to be clinically viable, they must move beyond excellent internal performance and undergo rigorous prospective validation to prove that the inclusion of such complex data actually improves clinical decision‐making and patient outcomes rather than merely increasing model complexity and the risk of overfitting.

### 4.2 Validity and Reliability

Most models demonstrated moderate to favorable predictive performance, with AUROC values typically ranging between 0.70 and 0.90 [49, 50, 51, 52]. A well‐calibrated model ensures that a predicted 20% risk of mortality truly corresponds to a 20% observed event rate, which is essential for clinical decision‐making. However, the reviewed studies rarely reported calibration metrics, and when they did, calibration was often suboptimal.

A critical concern remains the reported imbalance between sensitivity and specificity in some models. While a higher sensitivity may assist in identifying high‐risk patients, the accompanying lower specificity and potential for false‐positive predictions could lead to “alarm fatigue” or unnecessary clinical interventions, which are particularly risky in the complex management of ECMO patients. The tendency of models trained on retrospective data to exhibit miscalibration reflects both overfitting and the systematic differences between development and validation population.

Clinicians using these models should be aware that a ‘good’ AUROC does not guarantee that the model's probability estimates are reliable enough to guide individual patient decisions. Future efforts should prioritize the transparency of model limitations and the reporting of performance across diverse, independent cohorts to ensure that “excellent” metrics translate into safe and reliable clinical reliability.

### 4.3 Prospective Validation

Perhaps the most significant limitation of the current evidence base is the lack of prospective validation. Despite the encouraging retrospective performance reported across studies, no model has yet been evaluated in a prospective, real‐world clinical setting where its predictions are used to influence patient management. Retrospective validation, even when performed on held‐out test sets or external cohorts, cannot fully capture the complexities of clinical deployment: data drift over time, variations in documentation practices, and the interaction between model recommendations and clinician behavior. Consequently, the real‐world generalizability and clinical applicability of these models remain uncertain. Future research should therefore focus on prospective multicenter validation studies to confirm the reliability and clinical utility of AI‐based decision support systems before their routine integration into ECMO management.

### 4.4 Clinical Impact

AI‐based tools are currently best considered clinical decision‐support systems rather than independent decision‐making tools. Existing evidence suggests that AI may assist clinicians by improving risk stratification, early detection of complications, and optimization of resource utilization in ECMO programs [53, 54, 55].

However, direct evidence demonstrating improvement in patient‐centered outcomes—such as mortality, ICU length of stay, or quality of life—is currently lacking. For AI to realize its promise in ECMO, studies must move beyond model development and retrospective validation to demonstrate that AI‐guided interventions translate into measurable clinical benefits.

### 4.5 Future Perspectives and Methodological Challenges: Integrating Multimodal Data

A significant finding of this review is the current “underdevelopment” of multimodal AI integration in ECMO management, particularly concerning echocardiography and high‐frequency physiologic monitoring. Several methodological challenges identified in the reviewed studies warrant attention in future work. The lack of standardized definitions for outcomes such as “successful weaning” or “major bleeding” complicates model development and comparison.

This “information bottleneck” is likely due to the absence of unified data infrastructures capable of synchronizing high‐volume streaming data with discrete electronic health records. Moving forward, the transition from static “snapshot” models to dynamic, continuous‐learning systems that incorporate both bedside imaging and high‐frequency physiological signals is essential.

### 4.6 Limitations of the Reviewed Studies

The heterogeneity of ECMO modalities across the included studies (VA‑ECMO, VV‑ECMO, and ECPR) reflects the broad scope of artificial intelligence applications in extracorporeal support but may limit direct comparison of model performance across studies.

The lack of standardized definitions for outcomes such as “successful weaning“ or “major bleeding” complicates model development and comparison [23, 24].

Additionally, the reliance on the Extracorporeal Life Support Organization (ELSO) registry, while providing large sample sizes, introduces selection bias, as the registry excludes patients who were considered for ECMO but not initiated. Data quality challenges were consistently reported across studies, including missing values, inconsistent documentation, and variable definitions across centers. These issues not only complicate model development but also threaten the generalizability of findings [19]. Thus, further large multicenter trials are needed for external model validation of clustering and analysis of larger patient characteristics. Models trained on data from one region may not generalize to others due to differences in clinical practice, population genetics, and environmental factors.

Impacts of the model on direct clinical aspects have not been evaluated, and further study is needed before implementation in the field [37].

### 4.7 Limitations of This Review

This scoping review has several limitations that should be acknowledged. First, the reliance on published literature may introduce publication bias, as studies reporting favorable AI performance are more likely to be published than those with negative findings. Second, the heterogeneity of the included studies—in terms of AI models, outcome definitions, and ECMO populations—precluded quantitative meta‐analysis. Third, our quality assessment using the NOS revealed that the majority of studies were of moderate quality, primarily due to limitations in comparability and outcome assessment. Fourth, the rapid pace of AI development means that new models may have been published since our search date, and the field may have evolved beyond the evidence summarized here. Finally, while we attempted to identify gray literature and unpublished work, the search may not have captured all relevant studies, and our exclusion of non‐English publications may have introduced language bias.

### 4.8 Implications for Clinical Practice and Policy

Despite these limitations, several implications for clinical practice and policy emerge from this review. For clinicians, AI models should currently be viewed with cautious optimism—they may provide helpful risk estimates, but they should not replace clinical judgment, and their recommendations should be validated against local patient populations and institutional practices. For researchers, the findings highlight the urgent need for prospective, multicenter validation studies that evaluate not just predictive performance but also clinical utility and impact on patient outcomes. For policymakers and institutional leaders, investment in data infrastructure—including standardized data collection, integration of imaging and physiological waveforms, and interoperability between systems—is essential to enable the next generation of AI research in ECMO. Finally, for journal editors and reviewers, the promotion of standardized reporting guidelines (such as TRIPOD for prediction models) would enhance the quality, transparency, and reproducibility of research in this field.

## Author Contributions

**Zahra Asadi:** investigation, visualization, validation, writing – review and editing, data curation. **Mahmoud Shiri Kahnouei:** conceptualization, methodology, writing – review and editing, data curation. **Fathiyeh Bahramnejad:** validation, visualization, writing – original draft. **Alun C. Jackson:** conceptualization, writing – original draft, writing – review and editing. **Fatemeh Bahramnezhad:** methodology, writing – original draft, conceptualization, investigation, writing – review and editing, validation.

## Funding

The authors have nothing to report.

## Conflicts of Interest

The authors declare no conflicts of interest.

## Transparency Statement

Fatemeh Bahramnezhad, as the lead author and manuscript guarantor, affirms that this manuscript is an honest, accurate, and transparent account of the study being reported; that no important aspects of the study have been omitted; and that any discrepancies from the study as planned (and, if relevant, registered) have been explained.

## Supporting information


Supporting File 1

Supporting File 2

## Data Availability

The data that support the findings of this study are available on request from the corresponding author. The data are not publicly available due to privacy or ethical restrictions.
