# Supplementary material for: A Review of the Role of Artificial Intelligence in Patients on Extracorporeal Membrane Oxygenation: A Scoping Review Study
Source: Health Sci Rep. 2026 Aug 30;9(9):e73118. doi: 10.1002/hsr2.73118 (PMC13527396; doi:10.1002/hsr2.73118)
Supplement: Supplementary file 1 [file HSR2-9-e73118-s001.docx]

Supplementary File2: Methodological quality assessment of the included studies using the Newcastle-Ottawa Scale (NOS)

| Author(Year) | Selection | Comparability | Outcome | Total* | Quality Level |
| --- | --- | --- | --- | --- | --- |
| Morisson (2023) | ★★★ | ★ | ★★ | 6/9 | Moderate |
| Stephens (2023) | ★★★★ | ★★ | ★★★ | 9/9 | High |
| Shuo Wang (2024) | ★★★ | ★ | ★★ | 6/9 | Moderate |
| Abbasi (2020) | ★★ | ★ | ★ | 4/9 | Low |
| Shuai Wang (2025) | ★★★★ | ★ | ★★ | 7/9 | Moderate |
| Lee (2023) | ★★★ | ★ | ★★ | 6/9 | Moderate |
| Crespo-Diaz (2024) | ★★★ | ★ | ★★ | 6/9 | Moderate |
| Kim (2023) | ★★★ | ★ | ★★ | 6/9 | Moderate |
| Leng (2024) | ★★★ | ★ | ★★ | 6/9 | Moderate |
| Kamio (2025) | ★★★ | ★ | ★★ | 6/9 | Moderate |
| Fuller (2024) | ★★★ | ★ | ★★ | 6/9 | Moderate |
| Kalra (2023) | ★★★★ | ★★ | ★★★ | 9/9 | High |
| Liu (2023) | ★★★ | ★ | ★★ | 6/9 | Moderate |
| Alexander (2022) | ★★★ | ★ | ★★ | 6/9 | Moderate |
| Braun (2023) | ★★★ | ★ | ★★ | 6/9 | Moderate |
| Balian (2024) | ★★★★ | ★★ | ★★★ | 9/9 | High |
| Shou (2025) | ★★★ | ★ | ★★ | 6/9 | Moderate |
| Kresoja (2024) | ★★★★ | ★★ | ★★★ | 9/9 | High (RCT) |
| Hsu (2025) | ★★★★ | ★ | ★★ | 7/9 | Moderate |
| Ayers (2020) | ★★★ | ★ | ★★ | 6/9 | Moderate |

* Low Quality: 0-4 | Moderate Quality: 5-7 | High Quality: 8-9
