# Supplementary material for: A Review of the Role of Artificial Intelligence in Patients on Extracorporeal Membrane Oxygenation: A Scoping Review Study
Source: Health Sci Rep. 2026 Aug 30;9(9):e73118. doi: 10.1002/hsr2.73118 (PMC13527396; doi:10.1002/hsr2.73118)
Supplement: Supplementary file 2 [file HSR2-9-e73118-s002.docx]

| **Database** | **Search String** |
| --- | --- |
| **PubMed/MEDLINE** | (“Artificial Intelligence”[MeSH] OR “Machine Learning”[MeSH] OR “Deep Learning”[Title/Abstract] OR “Neural Networks”[Title/Abstract]) AND (“Extracorporeal Membrane Oxygenation”[MeSH] OR “ECMO”[Title/Abstract] OR “Extracorporeal Life Support”[Title/Abstract]) |
| **Scopus** | TITLE-ABS-KEY((“Artificial Intelligence” OR “Machine Learning” OR “Deep Learning” OR “Neural Network*”) AND (“ECMO” OR “Extracorporeal Membrane Oxygenation” OR “Extracorporeal Life Support”)) |
| **Web of Science** | TS=((“Artificial Intelligence” OR “Machine Learning” OR “Deep Learning” OR “Neural Network*”) AND (“ECMO” OR “Extracorporeal Membrane Oxygenation” OR “Extracorporeal Life Support”)) |
| **EMBASE** | (‘artificial intelligence’/exp OR ‘machine learning’/exp OR ‘deep learning’:ab,ti OR ‘neural network*’:ab,ti) AND (‘extracorporeal membrane oxygenation’/exp OR ‘ecmo’:ab,ti OR ‘extracorporeal life support’:ab,ti) |
| **National Databases** (SID, MagIran, IranMedex) | (“هوش مصنوعی” OR “یادگیری ماشین”) AND (“اکسیژن‌رسانی غشایی برون‌پیکری” OR “اکمو” OR “ECMO”) |

Supplementary File 1: Search strategy and syntax used across electronic databases.
